# A randomized, open-label pilot of the combination of low-level laser therapy and lorcaserin for weight loss

**DOI:** 10.1186/s40608-016-0122-4

**Published:** 2016-09-29

**Authors:** Ivana T. Croghan, Jon O. Ebbert, Darrell R. Schroeder, Ryan T. Hurt, Victoria Hagstrom, Matthew M. Clark

**Affiliations:** 1 Department of Medicine, Clinical Research Office, Clinical Trials Unit, Nicotine Research Program, Mayo Clinic, 200 First Street SW, Rochester, MN 55905 USA; 2 Center for the Science of Health Care Delivery, Mayo Clinic, 200 First Street SW, Rochester, MN 55905 USA; 3 Department of Health Sciences Research, Division of Biomedical Statistics and Informatics, Mayo Clinic, 200 First Street SW, Rochester, MN 55905 USA; 4 Department of Medicine, Division of General Internal Medicine, Mayo Clinic, 200 First Street SW, Rochester, MN 55905 USA; 5 A New Medspa Clinic, 3135 Superior Drive NW, Suite C, Rochester, MN 55901 USA; 6 Department of Psychiatry and Psychology, Mayo Clinic, 200 First Street SW, Rochester, MN 55905 USA

**Keywords:** Central adiposity, Lorcaserin, Low-level laser therapy, Obesity, Overweight, Weight loss

## Abstract

**Background:**

Obesity is a significant public health problem and innovative treatments are needed. The purpose of this pilot study was to assess the preliminary efficacy and safety of a combined treatment of low-level laser therapy (LLLT) and lorcaserin on weight loss, health quality of life (QOL) measures, and cardiovascular risk factors.

**Methods:**

Forty-five overweight and obese adult participants with a body mass index (BMI) >26.9 and <40 were randomized to receive LLLT, lorcaserin, or a combination of the two therapies. All study participants received treatment for 3 months and were followed for 3 months post-treatment. Participants were recruited from June 2014 through September 2014.

**Results:**

The majority of the 44 participants accrued to this study were female (84 %) with an average age of 43.9 years (range 22 to 64 years). Most participants (93 % LLLT alone, 87 % LLLT + lorcaserin) completed at least 80 % of the LLLT treatments. From baseline to end of treatment, significant reductions in waist circumference were noted for each treatment group (-2.3 ± 4.1 cm, -6.0 ± 7.3 cm, and -4.0 ± 5.5 cm for LLLT, lorcaserin and combination respectively); however, the reduction in body weight was only significant in those receiving lorcaserin and combination treatment (-0.4 ± 1.5 kg, -1.3 ± 1.2 kg and -1.3 ± 1.3 kg). No significant differences were noted between the groups. Self-reported satisfaction was higher in the lorcaserin versus the LLLT group.

**Conclusion:**

This small pilot demonstrates that when combined with behavioral intervention, Lorcaserin and LLLT may be effective components of a comprehensive approach to the treatment of overweight and obesity in the clinical setting. Further studies with larger sample size and longer duration of treatment and follow-up are needed to further address efficacy.

**Trial Registry Information:**

Trial registration: NCT02129608. Registered June 15, 2014.

## Background

In 2011–2012, approximately two-thirds of US adults were were either obese or overweight [1, 2]. Obesity significantly increases the risk for cardiovascular disease and is associated with poor quality of life (QOL). Weight reduction can modify both the risk and risk factors for cardiovascular disease [1, 3–6]. Weight reduction reduces blood pressure, triglycerides, and low-density lipoprotein (LDL) cholesterol; and increases high-density lipoprotein (HDL) cholesterol [7]. Reductions in waist circumference (WC) reduce the inflammatory biomarker C-reactive protein (CRP), LDL, diastolic blood pressure and overall cardiovascular risk [8].

Recent evidence has suggested that the distribution of adipose tissue (subcutaneous versus visceral) is more predictive of cardiovascular risk than body mass index (BMI) alone [9]. Central adiposity, which appears to be the best surrogate for estimating visceral adipose tissue, can be measured clinically as waist circumference (WC) and waist-to-hip ratio (WHR). Two large case–control studies have demonstrated that WC and WHR are independent risk factors for cardiovascular mortality [9, 10]; and measuring body composition is an important component of an effective weight loss program [11].

Unfortunately many obesity treatments are associated with poor adherence and high recidivism. Combining treatment modalities with different mechanisms of action to facilitate losing weight may hold the greatest potential for achieving meaningful weight loss, as individuals are provided with several approaches to successful weight management. Low-level laser therapy (LLLT) is a noninvasive body-contouring procedure designed to remove excess fat without emitting heat, sound, or vibrations. Originally designed to improve wound healing, reduce edema, and relieve pain [12, 13], LLLT has been utilized as an adjunct in Lipolysis (a process for removing fatty tissue) [12, 14–16], and was approved by the FDA in 2010 for fat reduction [17]. At that time the FDA identified this generic type of device as “a device using low level laser energy for the disruption of adipocyte cells within the fat layer for the release of fat and lipids from these cells for non-invasive aesthetic use.” [18]. It has also been shown to accelerate repair, stimulate cell proliferation, and promote vascularization in injured tissues [19]. Energy from the lasers (1.2 to 3.6 Joules/cm^2^) is proposed to permeabilize adipocyte membranes, resulting in extravasation of intra-adipocyte lipids. Lorcaserin is a selective serotonin 2C (5-HT_2C_) receptor agonist. Activation of the 5-HT_2C_ receptor subtype in the hypothalamus increases pro-opiomelanocortin (POMC) production leading to weight loss through satiety [20–22]. Lorcaserin is FDA-approved for weight management [23] in individuals with a body mass index (BMI) of >27 kg/m^2^ (overweight) when accompanied by a weight-related health condition such as type 2 diabetes or high blood pressure, or in people with a BMI >30 kg/m^2^ (obese) [20–22].

The purpose of this study was to evaluate the safety, feasibility, and preliminary efficacy of LLLT alone or in combination with lorcaserin for reducing anthropomorphic meaures of obesity. Biomarkers and adherence to treatment were also measured to provide guidelines for potential future investigations.

## Methods

### Trial Design

This was an open-label clinical trial in which participants were randomized to one of three treatments: (1) Lorcaserin 10 mg by mouth twice a day for 12 weeks; (2) LLLT administered for one hour once a week for 12 weeks; or (3) combination therapy with lorcaserin and LLLT for 12 weeks.

### Setting

**Fig. 1:**
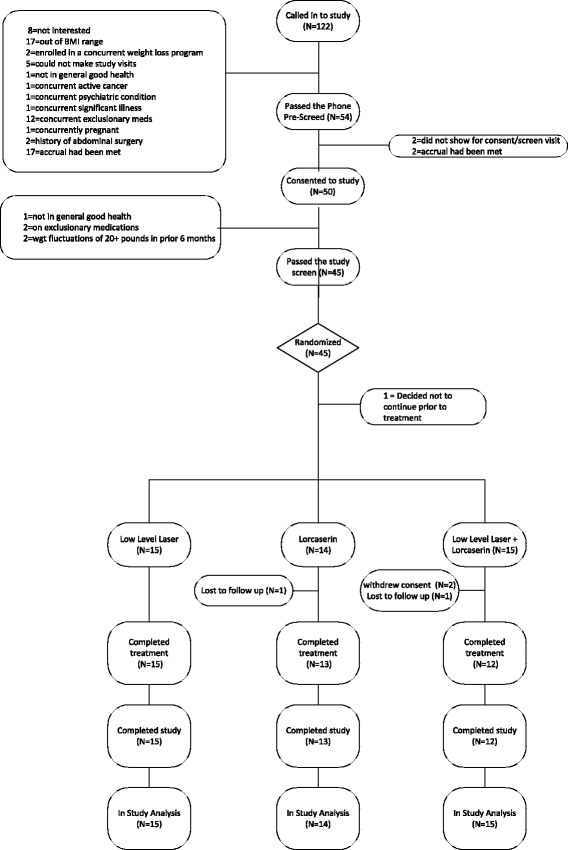
participant flow in study from first study contact to last study contact

A total of 45 participants were recruited from the local community of Rochester, MN from June 2014 through September 2014. Of these 45 individuals, with a BMI of 27–39.9 kg/m^2^, who were motivated to lose weight and enrolled in this study, 44 went on to receive study treatment. This report is based on the 44 participants who went on to be treated in study (Fig. 1). This consort diagram adheres to consort guidelines on reporting clinical trials [24].

### Participants

Study recruitment was through word of mouth (73 %), internet postings (24 %), and flyers (3 %). All interested individuals called a central number and underwent a 10-min phone pre-screen. If they passed the telephone pre-screen, they were invited to attend a one-on-one consent visit. After participants consented to be in study, they signed a written consent form and went on to be screened for study eligibility. If they passed the screening procedures they were invited to participate in the study. If they accepted the invitation, participants were randomized using a computer-generated randomization schedule. Study visits included a pre-screen phone interview, a combined consent/screen visit, a baseline visit at which the drug was dispensed and first laser treatment delivered, and a visit every other week during the first 12 weeks of study. Participants who received LLLT reported to the study office every week at approximately the same time and same day of the week. A phone visit was completed at week 13 (1 week after the end of treatment) to assess safety and a final study visit occurred at 6 months. This study was approved by the Mayo Clinic Institutional Review Board and written informed consent was obtained for all study particpants.

### Interventions

#### Low-Level Laser Treatment

The laser used in this study was an *Erchonia® Zerona™ 2.0 Laser.* This LLLT has been approved by the Food and Drug Administration (FDA) as a non-invasive dermatological aesthetic treatment to reduce the circumference of their hips, waist, and thighs (*Section 510(k) = K123237*). Each LLLT device consists of a multiple-head low-level diode laser with 6 independent diode laser heads. Each diode emits 532 nm (green) laser light. In the active LLLT [25] each diode generates a 17 milliwatt (mW) output. In this trial, participants engaged in weekly treatments for 12 weeks. Lasers were focused around the stomach and abdomen for 30 min and then aimed at the central region of their back for another 30 min. This was done once a week for 12 weeks for a total of 12 treatments. All study participants assigned to the laser were given instructions to wear body constricting undergarments during the 12 weeks of treatment and to drink plenty of fluids.

#### Lorcaserin

Participants were administred 10 mg orally twice daily for 12 weeks.

#### Behavioral Intervention

This study was dependent on successful behavior change, which in turn requires self-efficacy and motivation. Self-efficacy is defined as a sense of control over one’s ability to make the change [26, 27]. In this study we utilized a written self-help guide (*My Weight Solution*) to provide structure to the behavioral sessions consisting of a brief 5 to10 min one-on-one behavioral intervention at each study visit (every two weeks) based on different sections of the manual. Using a wellness coaching model [28], staff members personalized their message based on the participants progress and feedback during the study visit, this was dependent on the participants confidence and motivational level. When confidence was low, participants were encouraged to problem solve, use goal setting skills, seek social support, and think about their pass successes. When motivation was low, personal reasons for seeking to lose weight were reviewed to enhance motivation. Study staff received training and supervision from a weight management behavioral expert (MMC).

### Outcomes

Subjects’ motivation to reduce weight, follow a healthy diet, and maintain a physically active lifestyle were assessed at baseline prior to study interventions. Other assessments collected at baseline, end-of-treatment, and end-of-study included the following: (1) the Impact of Weight on Quality of Life-Lite (IWQOL-Lite) assessment, to assess self-perception on how weight affects daily physical health and emotional well-being [29, 30]; (2) the Linear Analogue Self-Assessment (LASA) to assesses QOL [31–33]; (3) the Body Areas Satisfaction Scale (BASS), a scale that is actually a sub-scale from the Multidimensional Body-Self Relations Questionnaire (MBSRQ) and used to assess patients’ self-perceived body image and satisfaction of 8 specific body areas [34–36]; and 4) the Body Appreciation Scale (BAS), assessment of positive body image [37]. Safety measures included adverse events information, concomitant medication information, and self report for despressive symptoms-reported depression using the Center for Epidemiologic Studies Depression Scale Revised (CESD-R). Biomarkers included blood leukocyte count, CRP, adipokines (leptin, adiponectin), cholesterol profile, HgbAlc and blood glucose. Anthropomorphic measures of weight, WC and hip circumference (HC) were collected. WHR were derived from the participants’ WC and HC. BMI and WHR were derived from measurement of weight, height, and waist and hip circumference. The measurements were as follows: *Height* of the subject in stocking feet were measured to the nearest 0.5 cm using a wall-mounted vertical stadiometer; *Weight* was measured wearing undergarments and no shoes to the nearest 0.1 kg by using a balance-beam scale calibrated weekly with certified weights. *BMI* was calculated using the standard formula of kg/m^2^; *WC* was measured by using a soft measuring tape placed in a horizontal plane around the abdomen midway between the lowest rib and the superior border of the iliac crest (the tape was snug but not compressing the skin and parallel to the floor with the measurement being made at the end of a normal expiration with the subject in an upright position); *HC* was measured at the maximal protrusion of the buttocks. Circumference was given as the mean of two measurements to the nearest 0.1 cm; *WHR* was calculated from the waist and hip measurements; *Adherence* to the study interventions was recorded as either attendance to the laser treatements and/or self report of pills taken per day on the subjects daily diaries, in addition to counting of pills dispensed and returned.

### Study Schedule

All subjects completed in person study visits every other week during the 12 weeks of treatment. This was followed by a safety phone call contact 1 week after the final treatment study visit and one final study visit at week 24. Vital signs and anthropomorphic measurements were collected at every study visits prior to any intervention, including behavioral intervention, adverse events, and concomitant medications. Study outcome assessments (IWQOL-Lite, LASA, BASS, BAS and CESD) and fasting biomarkers were collected at weeks 0 (baseline), 6, 12, and 24. During the final visit, satisfaction was measured via an end-of-study self-assessment survey. The 12 questions included “On a scale of 1 to 4, where “0” is not satisfied at all and “4” is extremely satisfied, how satisfied were you with your treatment assignment?”; other questions focused on the subjects’ perception of “effectiveness,” “usefulness,” and “difficulty.”

### Statistical Analysis

The primary endpoint was change in WC, per measurements taken at baseline and week 12. Based on preliminary data, the standard deviation for this endpoint was estimated to be 6.4 cm [38]. Although debate exists regarding the value of formal statistical comparisons in phase II trials, we agree with those who propose that a one-sided test with a false-positive (type I error) rate of 0.20 is an appropriate criterion to use to help guide the decision making process [39]. Under the assumption that a difference between groups of 4 cm or larger was clinically meaningful, we determined that a sample-size of *N* = 15 per group would provide statistical power (one-tailed, alpha = 0.20) of 80 % to conclude that future studies on combination therapy were warranted if a clinically meaningful difference exists.

In all cases, data were summarized using mean ± SD for continuous variables, and frequency percentages for nominal variables. Treatment adherence was quantified for each individual by calculating the percentage of treatment sessions attended and the percentage of medication used. Anthropomorphic measures at 12 weeks were compared between treatment groups using analysis of covariance (ANCOVA) with the baseline value included as the covariate. For these endpoints, the approach of last-value carried forward was used to impute values for subjects who discontinued study participation. The comparisons of combination therapy versus LLLT and lorcaserin monotherapies were of specific interest. For these comparisons, the treatment effect was reported using a point estimate and 90 % confidence interval. Similar analyses were performed for laboratory values and QOL measures. In all cases, distributional assumptions were assessed with transformations or nonparametric methods used as appropriate. In all cases, two-tailed p-values are reported. Study data were collected utilizing paper source case report forms. The data was managed using the REDCap tool hosted at Mayo Clinic. Data analyses were conducted using SAS statistical software (version 9.3, SAS Institute, Inc., Cary, NC).

## Results

A total of 122 individuals called with interest in this study,45 individuals were enrolled and randomized to study groups (15 LLLT, 15 lorcaserin, 15 combination therapy) and 44 went on to be treated according to their randomized assignment (15 LLLT, 14 lorcaserin, 15 combination therapy). This report is based on the 44 who went on to be treated in study (Fig. 1).

Baseline participant characteristics are presented in Table 1. The majority of participants were females (84 %), married or living as married (64 %), with at least some college education (95 %); their average age was 43.9 years (range 22 to 64 years). No significant differences were detected between the groups at baseline.

**Table 1:** Participant Characteristics

Characteristic	Overall (*N* = 44)	Treatment Group		
		LLLT (*N* = 15)	Lorcaserin (*N* = 14)	Combination (*N* = 15)
Age, years ± SD	43.9 ± 11.6	45.4 ± 9.9	40.6 ± 11.5	45.4 ± 13.3
Sex, N (%)				
Male	7 (16)	2 (13)	3 (21)	2 (13)
Female	37 (84)	13 (87)	11 (79)	13 (87)
Marital Status, N (%)				
Never married	5 (11)	1 (7)	1 (7)	3 (20)
Separated/divorced	9 (21)	3 (20)	2 (14)	4 (26)
Widowed	2 (4)	1 (7)	0 (0)	1 (7)
Married/living as married	28 (64)	10 (66)	11 (79)	7 (47)
Education, N (%)				
≤ High school graduate	2 (4)	0 (0)	2 (14)	0 (0)
Some college	25 (57)	10 (67)	7 (50)	8 (53)
≥ 4-year college degree	17 (39)	5 (33)	5 (36)	7 (47)
Weight at baseline, kg ± SD	93.5 ± 15.0	96.0 ± 15.3	94.4 ± 16.2	90.3 ± 14.0
Waist Circumference at baseline, cm ± SD	104.7 ± 12.6	105.3 ± 11.6	108.0 ± 15.7	100.9 ± 10.0

*SD* Standard Deviation

### Anthromporhic Measurements

At week 12, a significant decrease in WC from baseline to end-of-treatment (week 12) was observed for all treatment groups (-2.3 ± 4.1 cm, -6.0 ± 7.3 cm, and -4.0 ± 5.5 cm for the LLLT, lorcaserin, and combination groups, respectively; *P* < .05), as seen in Table 2. Significant reductions from baseline to week 12 were also observed for weight, BMI, and HC in the lorcaserin and combination groups. No significant differences were observed between treatment groups for any of the body measures assessed. At 6 months, the mean change in weight from baseline was negative for all treatment groups, and significantly different from baseline for the combination group (-1.4 ± 3.6 kg, *P* = .154; -2.4 ± 5.1 kg, *P* = .097; and -2.0 ± 3.5 kg, *P* = .045 for LLLT, lorcaserin, and combination groups, respectively). The change in weight from baseline to 6 months for the combination group was not significantly different from LLLT (*P* = .773) or lorcaserin (*P* = .775). The mean change in WC from baseline to 6 months was also negative for all treatment groups (-2.8 ± 4.3 cm, *P* = .023; -5.1 ± 8.8 cm, *P* = .051; and -2.8 ± 5.2 cm, *P* = .059 for LLLT, lorcaserin, and combination groups, respectively). The change in WC from baseline to 6 months for the combination group was not significantly different from LLLT (*P* = .942) or lorcaserin (*P* = .424).

**Table 2:** Anthropomorphic Measurements

Variable	Treatment group			Estimated treatment effect (90 % C.I.)^a^	
	LLLT (*N* = 15) ± SD	Lorcaserin (*N* = 14) ± SD	Combination (*N* = 15^b^) ± SD	Combination vs. LLLT only	Combination vs. Lorcaserin only
Weight, kg					
Baseline	96.0 ± 15.3	94.4 ± 16.2	90.3 ± 14.0		
Week 12	95.0 ± 14.2	90.7 ± 17.4	86.7 ± 14.2	−2.6 (-5.0, -0.3)	+0.1 (-2.3, +2.5)
Delta	−1.0 ± 4.4	−3.7 ± 3.3^c^	−3.5 ± 3.6^c^	*P* = .070	*P* = .950
Weight loss of >5 %, n (%)	2 (13)	8 (57)	5 (33)		
Body Mass Index, kg/m^2^					
Baseline	33.5 ± 3.9	33.6 ± 5.1	33.4 ± 4.2		
Week 12	33.1 ± 3.5	32.3 ± 5.7	32.1 ± 4.3	−1.0 (-1.8, -0.1)	+0.0 (-0.8, +0.9)
Delta	−0.4 ± 1.5	−1.3 ± 1.2^c^	−1.3 ± 1.3^c^	*P* = .063	*P* = .973
Waist Circumference, cm					
Baseline	105.3 ± 11.6	108.0 ± 15.7	100.9 ± 10.0		
Week 12	103.0 ± 11.3	102.0 ± 16.3	96.9 ± 12.4	−1.8 (-5.4, +1.7)	+1.7 (-1.9, +5.4)
Delta	−2.3 ± 4.1^d^	−6.0 ± 7.3^c^	−4.0 ± 5.5^d^	*P* = .398	*P* = .436
Hip Circumference, cm					
Baseline	119.1 ± 8.8	120.7 ± 12.1	119.0 ± 8.8		
Week 12	118.1 ± 8.3	116.2 ± 12.0	116.0 ± 9.3	−2.0 (-3.6, -0.3)	+1.5 (-0.2, +3.2)
Delta	−1.0 ± 3.1	−4.5 ± 2.1^e^	−3.0 ± 3.0^c^	*P* = .059	*P* = .159
Waist-to-Hip ratio					
Baseline	0.885 ± 0.086	0.892 ± 0.072	0.850 ± 0.063		
Week 12	0.874 ± 0.086	0.874 ± 0.087	0.834 ± 0.068	−0.007 (-0.032, +0.018)	−0.001 (-0.027, +0.025)
Delta	−0.011 ± 0.035	−0.017 ± 0.053	−0.016 ± 0.032	*P* = .657	*P* = .961

*CI* Confidence Interval, *SD* Standard Deviation

^a^Estimated treatment effect from analysis of covariance (ANCOVA) with the baseline value of the given variable included as a covariate

^b^There were 2 subjects in the combination group who discontinued prior to week 12 (one after week 2 and one after week 6). For these 2 subjects the last value of each variable was carried forward to week 12

^c^ *p* < 0.01 for paired *t*-test comparing week 12 versus baseline

^d^ *p* < 0.05 for paired *t*-test comparing week 12 versus baseline

^e^ *p* < 0.001 for paired *t*-test comparing week 12 versus baseline

### Quality of Life

The QOL measures at baseline and week 12 are summarized in Table 3. Overall QOL, as measured by LASA, improved significantly in those assigned to the LLLT group and combination therapy and the overall QOL as measured by IWQOL improved significantly in those assigned to combination therapy. In the lorcaserin group, self-perception of body image (BASS) and body appreciation scale (BAS) improved between baseline and week 12. Changes from baseline did not differ significantly across treatment groups for any of the QOL or body image/appreciation measures (*P* > .05).

**Table 3:** Quality of Life measures

Variable	Treatment Group		
	LLLT (*N* = 15)	Lorcaserin (*N* = 14)	Combination (*N* = 13^a^)
LASA ± SD			
Baseline	8.0 ± 0.9	7.7 ± 1.8	7.4 ± 1.1
Week 12	8.6 ± 0.9	8.6 ± 0.8	8.2 ± 1.3
Delta	+0.6 ± 0.9^b^	+0.9 ± 1.9	+0.8 ± 1.2^b^
CES-D ± SD			
Baseline	2.0 ± 3.6	4.1 ± 6.6	2.8 ± 3.3
Week 12	3.3 ± 4.4	3.7 ± 4.3	3.4 ± 2.8
Delta	+1.3 ± 2.5	−0.4 ± 4.8	+0.6 ± 2.8
BAS ± SD			
Baseline	3.7 ± 0.6	3.7 ± 0.5	3.7 ± 0.7
Week 12	3.6 ± 0.5	3.9 ± 0.7	3.8 ± 0.8
Delta	−0.1 ± 0.6	+0.3 ± 0.4^b^	+0.1 ± 0.7
BASS ± SD			
Baseline	3.0 ± 0.4	3.0 ± 0.5	3.0 ± 0.4
Week 12	3.1 ± 0.3	3.3 ± 0.5	3.0 ± 0.8
Delta	+0.1 ± 0.4	+0.3 ± 0.4^b^	0.0 ± 0.6
IWQOL - TOTAL ± SD			
Baseline	54.1 ± 12.1	50.9 ± 15.3	62.8 ± 22.4
Week 12	47.9 ± 7.4	47.6 ± 15.6	54.9 ± 21.9
Delta	−6.1 ± 10.4	−3.3 ± 8.7	−8.0 ± 7.9^c^

*BAS* Body Appreciation Scale, *BASS* Body Area Satisfaction Scale, *CESD-R* Center for Epidemiologic Studies Depression Scale Revised, *IWQOL-Lite* Impact of Weight on Quality of Life - Lite, *LASA* Linear Analogue Self-Assessment, *SD* Standard Deviation

^a^Of the 15 subjects assigned to the combination group, 2 discontinued prior to week 12 and are not included in the analysis

^b^ *P* < 0.05 for paired *t*-test comparing week 12 versus baseline

^c^ *P* < 0.01 for paired *t*-test comparing week 12 versus baseline

### Biomarkers

In the LLLT monotherapy, a significant decline between baseline and end of treatment occured in white blood cells and neutrophils. A significant decrease in triglycerides was observed in the lorcaserin monotherapy. In the combination therapy group, significant decreases in neutrophils and total cholesterol were observed. No differences were observed between groups (*P* > .05). Tables 4 and 5 provide the biomarker data.

**Table 4:** Lab values

Variable	LLLT (*N* = 15)	Lorcaserin (*N* = 14)	Combination (*N* = 13^a^)
RBC			
Baseline	4.59 ± 0.32	4.75 ± 0.43	4.80 ± 0.41
Week 12	4.61 ± 0.29	4.70 ± 0.42	4.65 ± 0.34
Delta	+0.01 ± 0.20	−0.06 ± 0.17	−0.15 ± 0.26
Hemoglobin			
Baseline	13.77 ± 0.92	14.34 ± 1.26	14.00 ± 1.26
Week 12	13.83 ± 0.81	14.08 ± 1.28	13.55 ± 1.15
Delta	+0.07 ± 0.57	−0.26 ± 0.61	−0.45 ± 0.86
Hematocrit			
Baseline	41.35 ± 2.07	42.81 ± 3.00	42.17 ± 2.82
Week 12	41.53 ± 2.13	42.03 ± 2.92	40.78 ± 2.67
Delta	+0.17 ± 1.82	−0.77 ± 1.93	−1.39 ± 2.37
MCV			
Baseline	90.19 ± 4.38	90.21 ± 3.18	88.09 ± 4.07
Week 12	90.29 ± 3.69	89.66 ± 3.76	87.86 ± 4.00
Delta	+0.10 ± 2.05	−0.56 ± 2.08	−0.23 ± 1.86
RDW			
Baseline	13.23 ± 0.74	13.11 ± 0.50	13.41 ± 0.61
Week 12	13.19 ± 0.58	12.96 ± 0.49	13.22 ± 0.69
Delta	−0.04 ± 0.40	−0.14 ± 0.37	−0.19 ± 0.48
WBC			
Baseline	6.83 ± 1.71	7.20 ± 1.45	6.98 ± 1.28
Week 12	5.99 ± 1.41	6.81 ± 2.11	6.32 ± 1.57
Delta	−0.83 ± 1.26^*^	−0.39 ± 1.08	−0.66 ± 1.11
Neutrophils			
Baseline	4.03 ± 1.58	4.15 ± 1.12	4.29 ± 1.08
Week 12	3.30 ± 1.10	3.99 ± 1.72	3.78 ± 1.20
Delta	−0.74 ± 1.17^*^	−0.16 ± 0.99	−0.51 ± 0.77^*^
Lymphocytes			
Baseline	2.04 ± 0.41	2.22 ± 0.55	2.00 ± 0.39
Week 12	1.93 ± 0.38	2.03 ± 0.41	1.84 ± 0.53
Delta	−0.11 ± 0.25	−0.19 ± 0.43	−0.16 ± 0.39
Monocytes			
Baseline	0.51 ± 0.13	0.60 ± 0.19	0.49 ± 0.13
Week 12	0.47 ± 0.13	0.58 ± 0.22	0.47 ± 0.10
Delta	−0.05 ± 0.12	−0.02 ± 0.12	−0.03 ± 0.06
Eosinophils			
Baseline	0.22 ± 0.12	0.18 ± 0.11	0.16 ± 0.05
Week 12	0.26 ± 0.18	0.17 ± 0.11	0.19 ± 0.15
Delta	+0.04 ± 0.11	−0.01 ± 0.06	+0.03 ± 0.15
Basophils			
Baseline	0.03 ± 0.02	0.04 ± 0.03	0.03 ± 0.02
Week 12	0.04 ± 0.02	0.04 ± 0.03	0.03 ± 0.01
Delta	+0.01 ± 0.02	−0.00 ± 0.02	+0.00 ± 0.01
Platelets			
Baseline	251.4 ± 56.6	287.0 ± 69.3	292.9 ± 60.3
Week 12	253.4 ± 51.2	277.1 ± 70.9	287.4 ± 47.7
Delta	+2.0 ± 20.8	−9.9 ± 27.3	−5.5 ± 26.0

^*^p < 0.05, for paired *t*-test comparing week 12 versus baseline

^a^Of the 15 subjects assigned to the combination group, 2 discontinued prior to week 12 and are not included in the analysis

**Table 5:** Lab values

Variable	LLLT (*N* = 15)	Lorcaserin (*N* = 14)	Combination (*N* = 13^a^)
Hgb_a1c			
Baseline	5.59 ± 0.92	5.31 ± 0.51	5.36 ± 0.23
Week 12	5.71 ± 1.12	5.29 ± 0.43	5.33 ± 0.30
Delta	+0.12 ± 0.29	−0.02 ± 0.14	−0.03 ± 0.13
CRP			
Baseline	4.72 ± 3.22	4.71 ± 3.62	4.60 ± 2.18
Week 12	4.81 ± 2.79	4.04 ± 2.55	5.97 ± 5.69
Delta	+0.09 ± 1.86	−0.68 ± 1.40	+1.37 ± 5.61
LDL			
Baseline	111.3 ± 28.7	95.1 ± 19.0	117.5 ± 31.0
Week 12	115.1 ± 29.4	94.9 ± 20.8	107.0 ± 28.8
Delta	+3.8 ± 22.2	−0.3 ± 17.8	−10.5 ± 22.2
Non-HDL Cholesterol			
Baseline	137.5 ± 33.0	128.4 ± 27.2	141.8 ± 34.7
Week 12	139.5 ± 37.3	121.6 ± 30.7	128.7 ± 33.7
Delta	+2.0 ± 24.6	−6.8 ± 18.8	−13.1 ± 24.2
HDL			
Baseline	62.3 ± 14.5	53.6 ± 14.5	60.3 ± 16.7
Week 12	61.5 ± 17.5	51.3 ± 12.7	56.5 ± 15.7
Delta	−0.8 ± 7.5	−2.3 ± 7.1	−3.8 ± 7.7
Triglycerides			
Baseline	130.7 ± 48.7	166.1 ± 98.5	119.8 ± 51.5
Week 12	121.9 ± 62.3	134.1 ± 85.3	108.7 ± 50.1
Delta	−8.9 ± 39.7	−32.1 ± 39.0^**^	−11.2 ± 56.2
Total Cholesterol			
Baseline	199.8 ± 29.0	182.0 ± 26.7	202.1 ± 27.3
Week 12	201.0 ± 35.7	172.9 ± 30.8	185.2 ± 33.0
Delta	+1.2 ± 30.1	−9.1 ± 23.5	−16.8 ± 24.2^*^
Fasting Glucose			
Baseline	89.7 ± 11.7	90.6 ± 14.6	88.6 ± 13.6
Week 12	92.5 ± 20.1	60.2 ± 17.8	87.7 ± 8.7
Delta	+2.8 ± 10.9	−0.4 ± 7.0	−0.9 ± 8.1
Leptin			
Baseline	38.1 ± 15.7	35.7 ± 21.2	37.2 ± 26.0
Week 12	34.0 ± 12.4	34.1 ± 23.6	30.8 ± 21.4
Delta	−4.1 ± 11.6	−1.6 ± 11.3	−6.4 ± 12.1
Adiponectin			
Baseline	9,719 ± 5,534	7,955 ± 3,463	12,412 ± 8,393
Week 12	8,987 ± 4,050	7,156 ± 2,231	12,254 ± 7,273
Delta	−732 ± 3,970	−799 ± 2,237	−158 ± 2,787

^*^ *p* < 0.05, ^**^ *p* < 0.01for paired *t*-test comparing week 12 versus baseline

^a^Of the 15 subjects assigned to the combination group, 2 discontinued prior to week 12 and are not included in the analysis

### Adverse Events

No serious adverse events (AEs) were observed for any of the assigned treatment arms. Ten AEs, occurring in the lorcaserin monotherapy and combination therapy groups, were reported by 6 individuals as being related to lorcaserin: dizziness (1 loracaserin, 1 combination), nausea (1 loracaserin, 2 combination), headache (1 loracaserin, 2 combination), low blood pressure (1 combination), and tingling sensation (1 combination). No AEs that “possibly,” “probably,” or “definitely” were related to LLLT were reported.

### Adherence

In the LLLT group, 93 % (14/15) attended at least 80 % of the treatments. In the lorcaserin group, 86 % (12/14) took at least 80 % of the dispensed doses. In the combination group, 73 % (11/15) took at least 80 % of the dispensed dose and 87 % (13/15) attended at least 80 % of the LLLT treatments. Among participants providing information at end of treatment, 50 % (7/14) in the LLLT group and 50 % (6/12) in the combination group reported complying with the body constricting undergarment recommendation; while 50 % (7/14), 69 % (9/13), and 58 % (7/12) (LLLT, lorcaserin, and combination groups, respectively; *P* = .605) indicated that they increased their water intake.

Among the 3 treatment groups (LLLT, lorcaserin, and combination therapy), 29 % (4/14), 54 % (7/13), and 75 % (9/12), respectively, indicated that they reduced their caloric intake *(P* = .063); 64 % (9/14), 85 % (11/13), and 92 % (11/12) indicated that they made dietary modifications (*P* = .224); and 43 % (6/14), 54 % (7/13), and 58 % (7/12) indicated that they increased their physical activity level (*P* = .784).

### Satisfaction

Overall satisfaction with the program was reported as “satisfied” or “extremely satisfied” by 29 % (4/14) in the LLLT group, 100 % (13/13) in the lorcaserin group, and 92 % (11/12) in the combination group (*P* < .001).

## Discussion

In this pilot project examing combined therapy for weight management, LLLT monotherapy, lorcaserin monotherapy, and combination therapy significantly reduced anthropomorphic measurements among overweight and obese adults. These interventions were also associated with improvements in body satisfaction and QOL. No side effects were reported related to LLLT and few side effects were reported related to lorcaserin. Satisfaction was higher with lorcaserin monotherapy and combination compared to LLLT alone.

Although no significant differences were observed between groups, consistent with previous literature, all three interventions were associated with reductions in at least one anthroporphic measurement. In a double-blind, placebo-controlled study (active vs. “sham” LLLT), 67 overweight patients were randomized to LLLT (17 mW) or sham-LLLT (2.5 mW) and received 6 treatments over 2 weeks. Sixty-three percent (22/35) of subjects in the LLLT group participants achieved individual success, defined as at least 3.0 in. reduction in combined circumference measurements for waist, hip, and bilateral thighs from baseline to after treatment phase completion, compared with 6 % (2/32) of participants in the sham group [40]. A larger study (*N* = 689) from the same investigative team indicated that the reductions in circumference were not attributable to fluid loss or fat relocation [41]. Retrospective studies have supported the body measurement reductions achieved (e.g., waist, hips, thighs) with this therapy and reported concomitant decreases in weight [42].

The treatment and efficacy of lorcaserin for weight loss has been evaluated in three large phase III clinical trials, with over 8,000 overweight and obese subjects, in which a weight loss of 3 % to almost 4 % was found at 1 year. The Behavioral Modification and Lorcaserin for Overweight and Obesity Management (BLOOM) trial enrolled 3,182 subjects and observed an average weight loss of 5.8 ± 0.2 kg with lorcaserin and 2.2 ± 0.1 kg with placebo during year 1 (*P* < .001) [20]. The Behavioral Modification and Lorcaserin Second Study of Obesity Management (BLOSSOM) trial enrolled 4,008 patients and showed that more people lost at least 5 % of their body weight at one year compared to placebo (47 %, 40 %, vs. 25 %; *P* < .001, lorcaserin twice daily, lorcaserin once daily, vs. placebo, respectively) [21]. The Behavioral Modification and Lorcaserin for Obesity and Overweight Management in Diabetes Mellitus (BLOOM-DM) trial, which enrolled 604 obese/overweight individuals with diabetes observed that more patients lost ≥5 % body weight with lorcaserin twice daily (38 %; *P* < .001) or lorcaserin daily (45 %; *P* < .001) compared with placebo (16 %) [22]. In our study, we observed an average weight loss of 3.5 to 3.7 kg (combination and lorcaserin group, respectively) by end of 12 weeks of treatment; whereby, similarly to these past studies, 57 % (8/14) in the lorcaserin group and 33 % (5/15) in the combination therapy lost at least 5 % or more of their baseline weight by end of treatment.

Because QOL is so important and many obese adults report having a negative QOL [43], it is crucial to assess the QOL of participants in weight-loss programs as it has been reported that weight loss can improve QOL [44]. In this study we observed that participants in the LLLT conditions improved their QOL, which suggests that there may be an important psychosocial dimension of this treatment. We hypothesize that participants may have anticipated that the LLLT would improve their health and well-being. Given the importance of QOL to the individual, further exploration of this QOL finding is warranted.

Unlike the previous LLLT studies, in this study satisfaction with LLLT was low; we hypothesize that this may be related to unrealistically high expectations surrounding its utility. Several participants expressed dissatisfaction in not receiving LLLT and one participant went as far as refusing to continue with the study after finding out that her randomized assignment was lorcaserin monotherapy. Less than half of the subjects assigned LLLT adhered with the behavioral intervention instructions related to their behavior changes (i.e., wearing the restrictive undergarments during the treatment weeks and drinking plenty of water) leading the investigators to hypothesize that, as unrealistic as it seems, study participants assumed that the LLLT would take care of their weight loss with little or no lifestyle changes on their part.

The behavior change phenomenon of setting unrealistic expectations and becoming disappointed when those expectations are not met is not unusual in weight loss programs where setting unrealistic expectations is a barrier to actual weight loss [45]. For example, a study of 60 obese females (mean BMI of 36.3 kg/m^2^), where subjects set a goal of 32 % reduction in body weight the average weight loss was a little less than 17 % from their baseline weight and the subjects deemed the program a failure [46]. Two other studies with individuals who had a BMI of 40 kg/m^2^ or higher also found this same phenomenon [47, 48]. Leading us to conclude that individuals with higher baseline weight may have more unrealistic goals for weight loss and high unrealistic expectations set prior to the program lead to poor compliance with the program and worse outcomes [45]. This phenomenon was also observed in our study, where subjects expected a large weight loss while not adhering to the behavioral interventions provided by study staff, indicating that in future studies behavioral intervention should include an initial intake discussion whereby subjects can set realistic expectations with the study staff in order for the study to have the most impact. Individuals trying to lose weight should receive valid information at this time about the expected outcomes of the intervention they are receiving and this, in turn, may improve adherence with the program.

The small sample size limited the ability to detect significant differences between groups. The open label design limits our study due to patient selection bias [49], participant retention bias [50], and participant performance bias [51]. In this study, participants who entered the study had a preference for LLLT. We observed that although they had no choice in what they were assigned, some subjects dropped out of the study after a few treatments, if it was not the treatment they wanted; while we hypothesize that other subjects did not adhere to the behavioral changes recommended if they received the intervention they did not expect/prefer, we did not track adherence to preferences and cannot confirm this at this time.

Another potential limitation was the LLLT administration. Both in a clinical setting and in research trials, LLLT can be provided at three different frequencies: 40 min (20 min on each side-front and back 3 times a week (every 48 h); 60 min (30 min on each side) 2 times a week; and 60 min (30 min on each side) once a week. The contemporary theory postulates that the lysing of fat cells should be continuous and the fat cells should not be allowed to recover. When we designed the study we sought expert opinion and it was recommended that if we increase the duration from 20 min on each side to 30 min on each side, once a week would be adequate. Taking into consideration the participant burden and study attrition, we decided that this preliminary study would focus on the last option (once per week). While reducing the visits to once a week may have led to improved treatment adherence, we suspect that the treatment of the LLLT was not frequent enough to result in clinical benefit. The study outcomes did not substantiate that this was the correct decision. Another study is currently being designed to determine the best frequency, which will address the lysing of cells and the subject burden.

In order to open recruitment to a larger number of individuals, the BMI inclusion criteria was expanded to include anyone with a BMI of 27–39.9, which included those categorized as overweight (BMI 25–29.9), obese level 1 (BMI 30–34.9), and obese level II (BMI 35–39.9). At the time that the study started, only the *Zerona™ 2.0 LLLT* option was available for anyone with a BMI of ≥25. After completion of the study, a second type of LLLT was approved by the FDA. Two LLLTs are now approved by the FDA for body sculpting: a 6-headed green laser (K130922) for adults with a BMI of 25–29.9 kg/m^2^ (NCT01702259) and a 10-headed green laser (K142042) for patients with a BMI of 30–40 kg/m^2^ (NCT01821352). If indeed the 10-headed green laser provides greater treatment effect for those individuals with a BMI between 30–40 kg/m^2^, as the marketing implies, then we are undertreating our patients who fall in this range.

Another potential limitation is related to variability in the behavioral counseling and lack of tailoring of the behavioral intervention to LLLT. Since LLLT is a new weight loss intervention it is not known if healthy lifestyle recommendations should be tailored for LLLT, such as more focus on strength training, use of form fitting clothing, or nutritional guideline. In terms of the variability of the behavioral intervention, in order to accommodate the study participants, a large coordinating staff was used. No study participant was guaranteed the same staff member at all visits. While, the study staff was uniformly trained on the behavioral intervention approach, the behavioral intervention had to target the subjects personal needs and therefore varied between subjects and staff members. As a result, the counseling approach varied depending on the staff member. In addition, the study staff was trained using an established protocol for collecting body measurements. We did not perform a formal study to assess variability of the measurement process. Although variability in the measurement process contributes to the variability seen in body measurements, each study subject was seen by different staff throughout the course of the study and therefore the variability of the measurement process should not introduce any bias when comparing across treatments*.*

Future studies will need to consider another approach to providing uniform counseling (e.g., identifying key behavioral topics for each session, or the use of online programs), collecting body measurements uniformally (e.g., iDEX), and investigating potential tailoring of a behavioral weight management intervention to LLLT.

## Conclusion

While there has been some concern regarding the safety of LLLT, in this small pilot study we did not find any indication of harmful effects based on the biomarkers or self-reported side effects, which provides some initial support for LLLT being a safe intervention. It appears that LLLT targets central adiposity, and our finding of reduction in waist circumference supports this premise. Further exploration utilizing a larger sample size with longer duration of treatment and follow-up is warranted to assess the safety and efficacy of LLLT in the treatment of obesity.

## Abbreviations

AE: Adverse Event
ANCOVA: Analysis of Covariance
BAS: Body Appreciation Scale
BASS: Body Areas Satisfaction Scale
BLOOM: Behavioral Modification and Lorcaserin for Overweight and Obesity Management
BLOOM-DM: Behavioral Modification and Lorcaserin for Obesity and Overweight Management in Diabetes Mellitus
BLOSSOM: Behavioral Modification and Lorcaserin Second Study of Obesity Management
BMI: Body Mass Index
CESD-R: Center for Epidemiology Studies Depression Scale - Revised
CRP: C-Reactive Protein
HC: Hip Circumference
HDL: High-Density Lipoprotein
IWQOL - Lite: Impact of Weight on Quality of Life - Lite
LASA: Linear Analogue Self-Assessment
LDL: Low-Density Lipoprotein
LLLT: Low-Level Laser Therapy
MBSRQ: Multidimensional Body-Self Relations Questionnaire
QOL: Quality of Life
WC: Waist Circumference
WHR: Waist-to-Hip Ratio
